# Supplementary material for: Multimodal imaging and functional analysis of the chick NMDA retinal damage model
Source: PLoS One. 2021 Sep 7;16(9):e0257148. doi: 10.1371/journal.pone.0257148 (PMC8423281; doi:10.1371/journal.pone.0257148)
Supplement: S1 File — (ZIP) [file pone.0257148.s004.zip › SD-OCT/C200727/015/D8-PWk2/C200727-015_OS_R_14_0_REGAVG0000027/Manual_PreDefineThreeByFourGridRadialRetina_1.pdf]

# Three By Four Grid Radial Retina Template Report

| Patient ID | C200727 | Name | See InVivoVue |
|---|---|---|---|
| Exam Date | See InVivoVue | Exam Time | See InVivoVue |
| Eye | OS | Averaged | N/A |
| Depth (mm) | 1.65 | Depth Samples | 1024 |
| Pitch (um/pix) | 14.000 x 2333.333 x 1.608 | Refractive Index | See InVivoVue |
| Scan Type | Radial 14.000mm x 14.000mm x 1.647mm ,<br>1000 x 6 x 1024 | | |

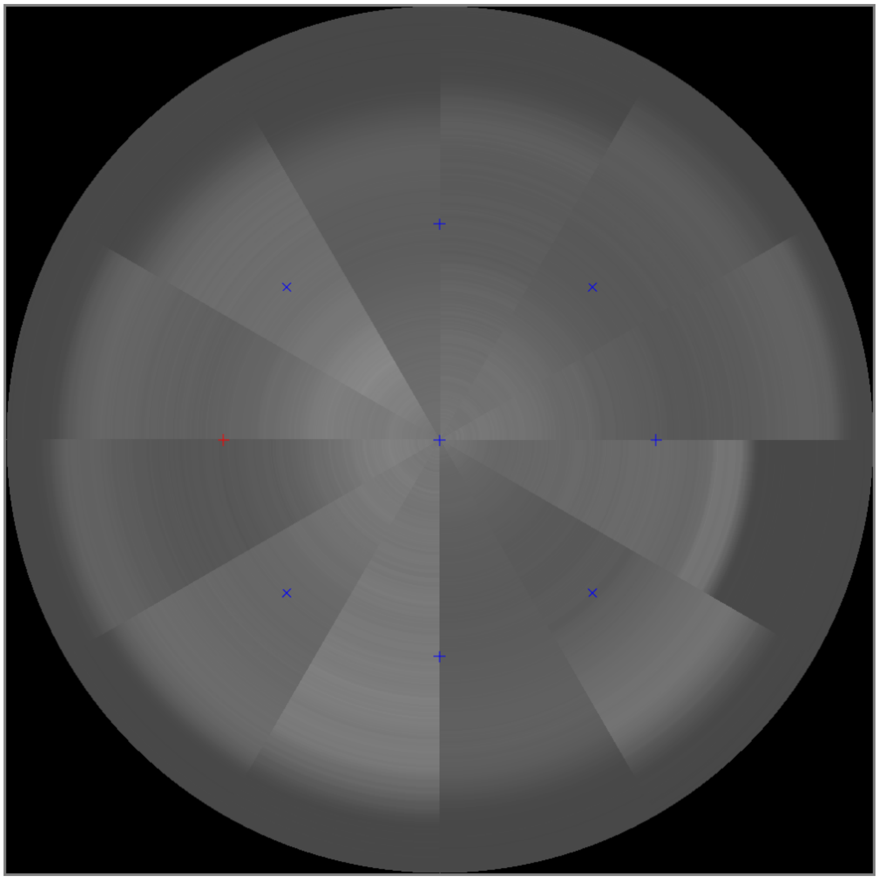

## Thickness Metrics

| Analysis | Average(mm) | Minimum(mm) | Maximum(mm) | STDEV(mm) |
|---|---|---|---|---|
| IRNFL - RPE | 0.2893 | 0.2790 | 0.3052 | 0.0108 |
| IRNFL - ORNFL | 0.0431 | 0.0206 | 0.0724 | 0.0203 |
| ORNFL - OPL | 0.1679 | 0.1479 | 0.1966 | 0.0160 |
| OPL - ETPRS | 0.0580 | 0.0512 | 0.0624 | 0.0034 |
